# Unified understanding of intrinsic and extrinsic controls of dissolved organic carbon reactivity in aquatic ecosystems

**DOI:** 10.1002/ecy.3763

**Published:** 2022-08-10

**Authors:** Martin Berggren, François Guillemette, Magdalena Bieroza, Ishi Buffam, Anne Deininger, Jeffrey A. Hawkes, Dolly N. Kothawala, Richard LaBrie, Jean‐François Lapierre, Kathleen R. Murphy, Enass S. Al‐Kharusi, Mayra P. D. Rulli, Geert Hensgens, Hani Younes, Urban J. Wünsch

**Affiliations:** ^1^ Department of Physical Geography and Ecosystem Science Lund University Lund Sweden; ^2^ Département des sciences de l'environnement Université du Québec à Trois‐Rivières Trois‐Rivières Québec Canada; ^3^ Groupe de recherche interuniversitaire en limnologie (GRIL) Montréal Québec Canada; ^4^ Department of Soil and Environment Swedish University of Agricultural Sciences Uppsala Sweden; ^5^ Department of Landscape Architecture, Planning and Management Swedish University of Agricultural Sciences Alnarp Sweden; ^6^ Norwegian Institute for Water Research (NIVA) Oslo Norway; ^7^ Centre for Coastal Research (CCR), University of Agder Kristiansand Norway; ^8^ Department of Chemistry BMC, Uppsala University Uppsala Sweden; ^9^ Department of Ecology and Genetics Uppsala University Uppsala Sweden; ^10^ Département des Sciences biologiques Université de Montréal Montréal Quebec Canada; ^11^ Interdisciplinary Environmental Research Centre Freiberg Germany; ^12^ Department of Architecture and Civil Engineering Chalmers University of Technology Gothenburg Sweden

**Keywords:** bioreactivity, dissolved organic carbon, photoreactivity, sorption

## Abstract

Despite our growing understanding of the global carbon cycle, scientific consensus on the drivers and mechanisms that control dissolved organic carbon (DOC) turnover in aquatic systems is lacking, hampered by the mismatch between research that approaches DOC reactivity from either intrinsic (inherent chemical properties) or extrinsic (environmental context) perspectives. Here we propose a conceptual view of DOC reactivity in which the combination of intrinsic and extrinsic factors controls turnover rates and determines which reactions will occur. We review three major types of reactions (biological, photochemical, and flocculation) from an intrinsic chemical perspective and further define the environmental features that modulate the expression of chemically inherent reactivity potential. Finally, we propose hypotheses of how extrinsic and intrinsic factors together shape patterns in DOC turnover across the land‐to‐ocean continuum, underscoring that there is no intrinsic DOC reactivity without environmental context. By acknowledging the intrinsic–extrinsic control duality, our framework intends to foster improved modeling of DOC reactivity and its impact on ecosystem services.

## INTRODUCTION

Dissolved organic carbon (DOC) undergoes numerous transformations as it flows from land to ocean, with both positive and negative effects on ecosystem services. Mineralization of DOC into greenhouse gases contributes to climate warming (Kosten et al., 2010; Tranvik et al., 2009) but also removes organic contaminants from potable water reservoirs (Bhatnagar & Sillanpää, 2017). Simultaneously, DOC that escapes mineralization may contribute to carbon sequestration and, thus, climate change mitigation, for example, via sedimentation (Battin et al., 2009). Thus, DOC turnover and fate are critical to both society and the global carbon cycle.

There are three main pathways of DOC transformation—biological reactions, sunlight‐induced photochemical reactions, and immobilization by flocculation—each controlled by the intrinsic chemical composition of the DOC but also by extrinsic physical, chemical, and biological factors (Anderson et al., 2019). However, despite recent advances in the conceptual understanding of large‐scale DOC turnover (Catalan et al., 2016; Raymond et al., 2016) and how to model it (Anderson et al., 2019), the combined intrinsic and extrinsic controls on these transformation pathways are inadequately described. Thus, the fates of DOC inputs from land to water in a changing environment remain unclear.

Here we review reactive organic matter features from a chemical perspective and further define the environmental conditions that contribute to corresponding DOC transformations. Based on this review, we formulate hypotheses of how extrinsic and intrinsic factors combined shape patterns in water column DOC turnover across gradients in the land–ocean continuum. Our framework aims to foster improved predictions of DOC reactivity and its impact on ecosystem services by showing that intrinsic DOC reactivity per se is a meaningless concept without the environmental context.

## REACTIVITY AND THE INTRINSIC–EXTRINSIC DUALITY

Reactivity is a broad and operational concept used to describe the rate of transformations. For DOC, it is typically measured as biological or photochemical mineralization rates in controlled conditions, but it can also refer to a physical reaction such as sorption to mineral surfaces. Reactivity is assessed on a continuous scale, which makes it different from the concept of *lability*, which categorizes DOC into classes of different reactivity potential (Guillemette & del Giorgio, 2011). Here we focus on reactivity as it describes the turnover rate at which DOC escapes the water column, i.e., through net loss processes, including mineralization and flocculation. We use the term DOC (carbon units) to clarify that reactivity of elements other than carbon (e.g., iron, phosphorus, nitrogen) is beyond scope of this paper (Berggren et al., 2015). This net turnover is a function of the dynamic array of intrinsic DOC chemistry (Mostovaya et al., 2016) and the extrinsic environments that facilitate potential DOC reactions (Anderson et al., 2019).

The dual intrinsic–extrinsic controls on DOC reactivity mean that reactive molecular features do not translate into DOC turnover if the extrinsic potential is lacking, and vice versa. For instance, a nutrient‐starved environment can protect an intrinsically bioreactive molecule like glucose from microbial degradation (Hessen et al., 1994), and a photoreactive compound will obviously escape photodegradation in darkness. However, because this dual control is largely ignored, inconsistencies emerge between studies that have an intrinsic versus extrinsic DOC reactivity perspective.

Intrinsically, most natural DOC has relatively low bioreactivity (Lapierre et al., 2013; Selvam et al., 2016) because of the inherent limitations in the affinity to enzymes (Mann et al., 2014). Conversely, a majority of DOC can be photodegraded (Köhler et al., 2002) or sorbed to mineral surfaces under appropriate conditions (Groeneveld et al., 2020). Nonetheless, when analyzed in natural settings, bioreactivity is a major cause of carbon turnover (Algesten et al., 2004; Anderson et al., 2019; Lapierre et al., 2013), whereas photo mineralization (Koehler et al., 2014) and flocculation (Anderson et al., 2019) are surprisingly minor in most cases, although there are exceptions (Molot & Dillon, 1997; Worrall & Moody, 2014). Thus, the inherent potential of most DOC to photodegrade or sorb is often not realized owing to environmental constraints, i.e., limited light availability and lack of surfaces to induce flocculation (Groeneveld et al., 2020).

Another conundrum is the relationship between photoreactivity and aromaticity, commonly approximated from specific UV absorption (SUVA). Intrinsically, aromatic compounds are expected to be highly reactive due to their efficient absorption of natural UV light (Maizel et al., 2017). However, though some field studies report strong positive correlations between photoreactivity and SUVA (Koehler et al., 2016), this correlation may be weak to absent (Cory et al., 2013) or even negative (Selvam et al., 2019) in other studies. This illustrates that intrinsic photoreactivity (as indicated by high SUVA) might not be expressed in the field, likely owing to extrinsic controls such as pH variations (Selvam et al., 2019). Thus, studies of intrinsic and extrinsic controls can provide strongly contrasting views on DOC reactivity, and yet the discrepancies between these approaches can provide deeper insight. There is a strong need to advance DOC reactivity research by considering intrinsic and extrinsic factors in synchrony.

## INTRINSIC REACTIVITY FROM A CHEMICAL FUNCTIONAL PERSPECTIVE

**FIGURE 1:**
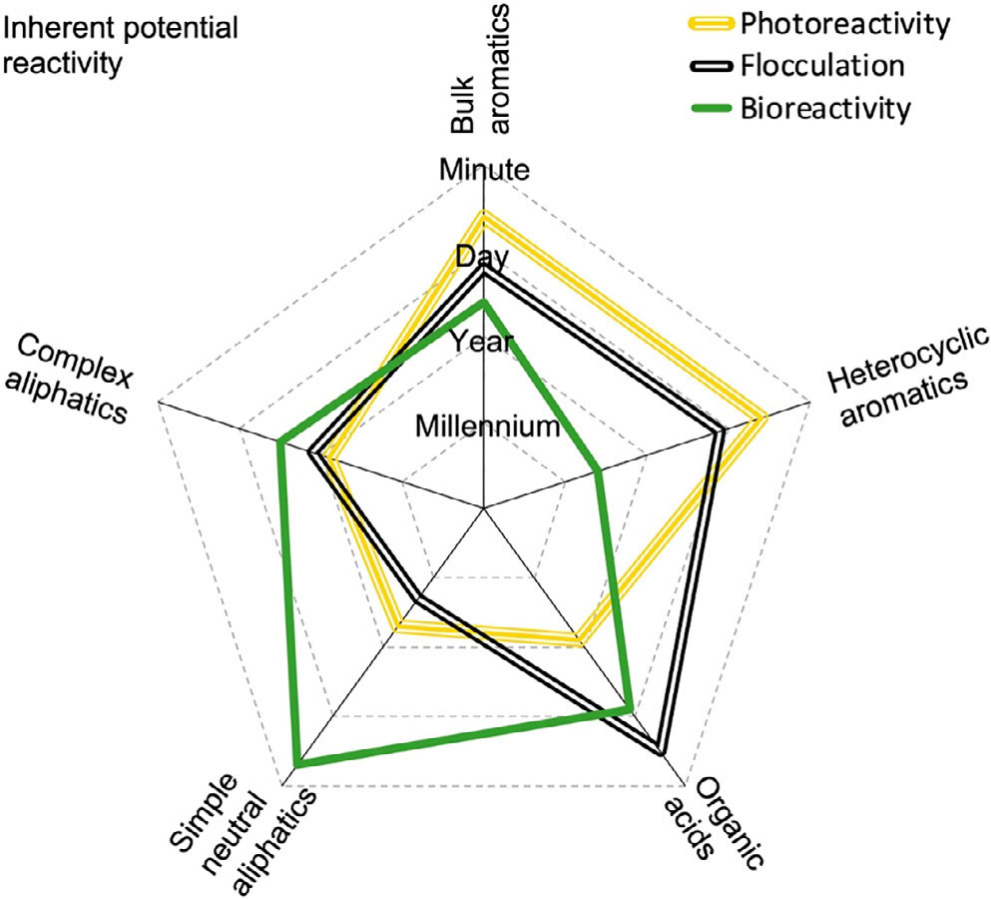
Simplified view on inherent reactivity profiles of dissolved organic carbon encountering three types of reactions (different lines) representing photoreactivity, flocculation, and bioreactivity relative to different structural and functional properties. The scale (gray dashed lines) shows inherent turnover potential, from millennia (innermost circle) to minutes (outermost circle).

Optical, isotopic, and molecular approaches provide information about the size, structure, and function of organic molecules and mixtures, as reviewed by McCallister et al. (2018). Studies of intrinsic reactivity indicate that aromatic molecules are generally reactive with light, whereas aliphatic and charged compounds are relatively more reactive to biological and sorption processes, respectively (Findlay & Sinsabaugh, 2003). A conceptual summary of inherent reactivity for different types of compounds is shown in Figure 1 and explained in what follows.

There are thousands of molecular formulas from dissolved organic compounds that are present at mostly trace concentrations (Brown et al., 2016; Hawkes et al., 2018) and that can each be assessed separately in terms of reactivity (Mostovaya, Hawkes, Koehler, et al., 2017). However, because compounds with different functional structures potentially share the same formula (Zark & Dittmar, 2018), functional information is needed as a complement. In this context, optical characteristics of the organic matter give qualitative information about key functional properties such as aromaticity (SUVA), whereas quantitative determination of functionality can be performed using nuclear magnetic resonance (NMR) (McCallister et al., 2018), in combination with mass spectrometry (Leenheer et al., 1995) or isotopic labeling techniques (Zherebker et al., 2017). Since reactions take place at the level of functional moieties rather than whole molecules, these functional approaches can shed light on the main DOC properties that are prone to take part in biological, photochemical, or sorption reactions.

### The chemistry of inherently bioreactive DOC

The turnover time of biological DOC compounds in aquatic systems (Figure 1) ranges from seconds or minutes for simple biomolecules such as amino acids, DNA, and ATP up to millennia for heterocyclic compounds (Amon et al., 2001; McCarthy et al., 1997). Nonetheless, most known molecular formulas have relatively slow biological turnover rates, with first‐order decay coefficients of 0.001–0.005 day^−1^, suggesting a turnover time on the scale of years (Mostovaya, Hawkes, Koehler, et al., 2017). Moreover, long‐term degradation experiments with lake water indicate that large parts of bulk DOC have a decade‐long half‐life (Koehler et al., 2012; LaBrie et al., 2020). Thus, the bulk of natural DOC is not readily biodegradable.

In general, small molecules are directly assimilable by microbes (Berggren et al., 2010; Nagata, 2008), whereas larger molecules require extracellular (exo‐) enzyme processing (Hoppe et al., 1988). Hence, low‐molecular‐weight sugars and organic acids are easily assimilated and typically highly bioreactive (Berggren et al., 2010), even if their nutritional and energetic qualities vary (del Giorgio & Cole, 1998; Vallino et al., 1996). However, small size is no guarantee of bioreactivity; small and abundant molecules of marine microbial origin are highly biorefractory, explaining observations of decreasing bioreactivity of bulk DOC along the freshwater to sea gradient (Amon & Benner, 1996). Moreover, large macromolecules are inherently biodegradable if they can be readily broken down into smaller molecules by extracellular enzymes, as is often the case for proteins, lipids, and polysaccharides (Nagata, 2008). Therefore, although molecular size constrains microbial assimilation of DOC, it is not a coherent indicator of bioreactivity.

With regard to functionality, a widely reported pattern is that carbon in aromatic rings, which typically constitute 10%–30% of aquatic DOC (McKnight et al., 2001), is less biodegradable than aliphatic organic carbon (Kalbitz et al., 2003; Qualls, 2004), although there are exceptions (Köhler et al., 2013; Mostovaya, Hawkes, Dittmar, & Tranvik, 2017). Bacteria cannot process large aromatic molecules such as lignin, in contrast to fungi (Higuchi, 2004). Nonetheless, the aromatic DOC pool includes significant fractions of compounds around 100 Daltons in size (Brown et al., 2016), which is small enough to be actively taken up and degraded by bacteria. Moreover, fungal preprocessing of large lignin‐like molecules may result in smaller assimilable molecules (Bonugli‐Santos et al., 2010). To conclude, there is a large variability in the reactivity of aromatics that remains to be defined chemically, but bioreactivity is relatively higher for simple aliphatics (Figure 1).

### Photochemically reactive inherent properties of DOC

Inherent photoreactivity potential is high for aromatic compounds but low for aliphatic DOC (Figure 1) because direct photoreactions are triggered by light‐absorbing features, primarily aromatic rings. Thus, shortwave radiation mineralizes DOC upon molecule‐photon interception. Additionally, reactive intermediate compounds are generated, including triplet‐excited organic molecules, reactive halogen species, and reactive oxygen species, such as hydroxyl radicals (McNeill & Canonica, 2016). These reactive intermediates trigger secondary reactions that further modify and mineralize DOC. For example, hydroxyl radicals can break down aromatic rings of molecules into organic acids (Waggoner et al., 2015), resulting in partially oxidized DOC that may (Allesson et al., 2016) or may not (Cory et al., 2013) be bioreactive. Photodegradation of DOC also produces a range of fluorescent secondary molecules (Murphy et al., 2018), some of which are easily biodegraded (Moona et al., 2021). Thus, photoreactions lead to a cascade of intramolecular rearrangements (McNally et al., 2005), which complicates the concept of photoreactivity.

Organic molecules with potential for direct photoreactions are defined by their light‐absorbing aromatic rings and double bonds, whereas the reactive properties of DOC involved in the secondary reactions vary with different photoproduced reactive intermediates and, thus, are less easily characterized. For example, the reactive oxygen species O_2_^−^·, ^1^[O_2_], and ·OH react with dissolved lignin species of the lowest, middle, and highest O:C ratios, respectively (Waggoner et al., 2017). According to McNally et al. (2005), the compounds that are generally most photorecalcitrant lack alpha‐carbonyl and phenolic functionalities, which implies that aromatic molecules are inherently most photoreactive despite being biorefractory (Figure 1).

### Inherent flocculation potential

Another pathway by which DOC is removed from the aquatic continuum is through flocculation, which is a major contributor to sedimentation (von Wachenfeldt & Tranvik, 2008). Although a fraction of flocculated particles is lost through mineralization (Attermeyer et al., 2018), flocculation is a first step toward permanent carbon burial. Overall, molecules with dense anionic functional features/moieties have high potential to aggregate and coprecipitate with positively charged interfaces upon collision (Kepkay, 1994). Additionally, organic molecules can be more or less likely to coaggregate with metal ions through sweep flocculation, for example, in iron‐rich lakes (Köhler et al., 2013). Thus, not all molecules are inherently likely to sorb onto particles.

Carboxylic acids are the main functional components that can act as ligands together with metals on, for example, clay (Kaiser & Guggenberger, 2000; Specht et al., 2000). However, once carboxylic acids have been sorbed, additional layers of more complex organic molecules, such as aromatics, sorb onto the surface in a secondary stage (Mitchell et al., 2018). Moreover, the high hydrophobicity of aromatic molecules makes them prone to flocculate through hydrophobic interactions even without mineral surfaces (Hakim & Kobayashi, 2018). Therefore, hydrophobic and charged molecules have an inherently high flocculation potential (Figure 1) compared to aliphatics. Overall, current evidence suggests that the three main reactivity pathways in aquatic environments tend to preferentially target different components of the DOC pool (Figure 1).

## EXTRINSIC ENVIRONMENTAL DRIVERS OF REACTIVITY

**FIGURE 2:**
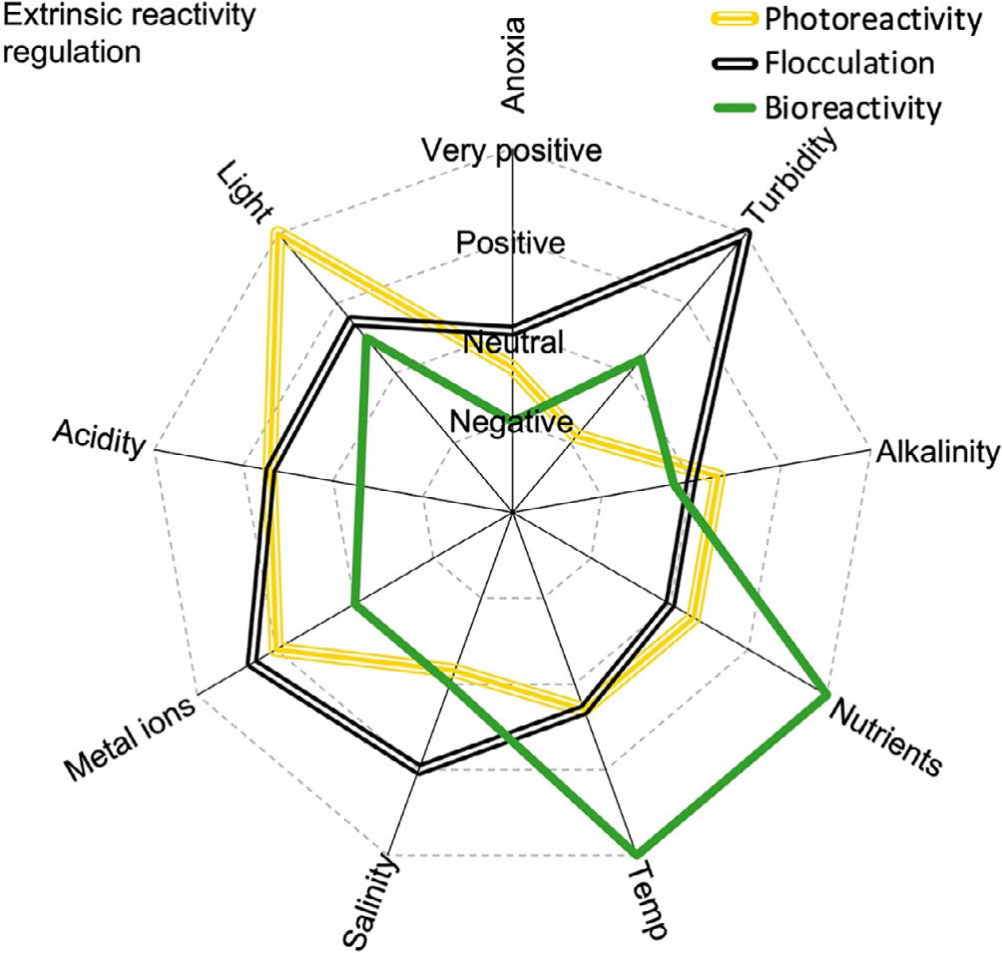
Simplified representation of relative impact of different extrinsic factors on dissolved organic carbon reactivity in the environment.

For any given inherent reactivity, DOC turnover rates in aquatic environments are strongly influenced by physical, biological and chemical drivers (Figure 2). As in soils (Schmidt et al., 2011), aquatic DOC reactivity can be viewed as an emerging ecosystem property constrained by its environment (Kothawala et al., 2021). In this context, we describe how encounters between DOC molecules and extrinsic factors (e.g., heat, light, O_2_, pH) are responsible for DOC removal.

### Temperature as a key extrinsic regulator of DOC turnover

All reactions that depend on kinetic energy are temperature dependent, which can be estimated from the activation energy term (*E*_a_) in the Arrhenius equation or by the *Q*_10_ factor, which describes the increase in degradation rate per 10°C increment. The temperature dependence of bioreactions is modulated by molecular features (Davidson & Janssens, 2006), but at the ecosystem level, *E*_a_ for aquatic bioreactivity measured as respiration is surprisingly constant at around 50–70 kJ mol^−1^ (Yvon‐Durocher et al., 2012) and *Q*_10_ of 2–3 at ~15°C (Carignan et al., 2000; von Wachenfeldt et al., 2009). This suggests that the relative impact of temperature on ecosystem‐scale DOC bioreactivity can be roughly predicted as an environmental effect without taking inherent chemical properties into consideration, at least in the case of bioreactivity.

Interestingly, temperature dependency is relatively weak for photoreactions and sorption with clay, with *Q*_10_ of 1.0–1.5 for both processes (Kaiser et al., 2001; Porcal et al., 2015), mathematically equivalent to *E*_a_ below ~30 kJ mol^−1^. A low *E*_a_ value of 0–33 kJ mol^−1^ was obtained in models of DOC loss from River Tees, England (Worrall & Moody, 2014), suggesting that photoreactions and/or flocculation dominated. In contrast, the modeled in situ DOC turnover in north‐temperate lakes had a higher *Q*_10_ of 2 typical for biological mineralization (Hanson et al., 2011). Thus, the temperature dependence of bulk DOC turnover in nature can give a qualitative indication of the dominant reactions.

### Extrinsic controls on bioreactivity

A well‐known extrinsic regulator of bioreactivity is the supply of essential nutrients, especially labile N and P macroelements (Berggren et al., 2015), which positively influence DOC turnover rates (Smith & Prairie, 2004; Soares et al., 2018). Therefore, in nutrient poor freshwaters with inherently labile carbon, for example, permafrost thaw streams, it is unsurprising that experimental N + P additions can double DOC turnover rates (Textor et al., 2019). However, in freshwaters with high colored organic matter content, bioreactivity is often carbon limited rather than N or P limited (Koehler et al., 2012; Soares et al., 2017). This is partly because the dissolved organic matter in brown‐water systems often supplies more bioavailable N and P than C, relative to bacterioplankton needs (Soares et al., 2017). Moreover, bacteria can shift metabolic balance from nutrient‐demanding growth to maintenance respiration, which requires fewer nutrients (del Giorgio & Cole, 1998; Jansson et al., 2006). Therefore, microbial DOC turnover rates can be maintained at relatively high rates, even at low inorganic nutrient concentrations.

The composition and functional structure of aquatic microbial communities quickly respond to environmental changes (e.g., changing salinity or pH) and may reach near‐optimum capacity for bulk DOC turnover within days (Judd et al., 2006; Logue, Stedmon, et al., 2016). However, it takes years of residence time until the microbial community composition fully stabilizes (Lindström et al., 2006). The order and timing of the decay of different compounds, that is, which are used first (Logue, Stedmon, et al., 2016) and the biochemical decay pathways (Comte & del Giorgio, 2011) vary widely as functions of microbial community composition. With increasingly extreme environments (e.g., extremely high salinity or temperature) it is more likely that DOC bioreactivity depends on specific taxa such as archaea (Logue, Findlay, et al., 2016), for example, organisms living in anoxic hypolimnia tend to degrade DOC slowly (Bastviken et al., 2004). Thus, to understand aquatic bioreactivity, it is important to know how the environment influences the presence and functions of decomposers.

Extrinsic controls on bioreactivity include factors in the environment that influence DOC losses by stressing bacteria. However, there is no consensus on the influence of viral activity (Bonilla‐Findji et al., 2008), bacterivory (Bana et al., 2014), toxins (Pringault et al., 2016), or salinity (Chinleo & Benner, 1992; Langenheder et al., 2003) on bacterial carbon mineralization rates, since they all can have variable and sometimes positive effects. Extremely high (>10) or low (<4) pH values strongly limit bacterial metabolism, but any pH value can have a negative effect if the community is not adapted to it (Bååth & Kritzberg, 2015). Interestingly, a stress factor that is associated with increased bioreactivity is UV light, which converts biorefractory compounds into labile DOC (Ruiz‐González et al., 2013). Thus, a stressor may still result in a net positive bioreactivity through its influence on organic matter composition.

In summary, extrinsic DOC bioreactivity is generally dominated by positive effects from temperature and nutrients, but lack of oxygen may have large negative impacts (Figure 2).

### Extrinsic controls on photoreactions

Rates of direct photochemical reactions scale in proportion to incoming solar UV light, which is affected by extrinsic factors like sun angle, cloud cover, ozone layer thickness, and shading effects of particles and colored substances (Koehler et al., 2014; Worrall & Moody, 2014). Indirect photodegradation by reactive oxygen species tends to increase with DOC concentration (Murphy et al., 2018) but also depends on many other factors. For example, nitrite and nitrate cause photolytic release of hydroxyl radicals that, in turn, react with DOC (Zepp et al., 1987), and iron catalyzes radical formation through a series of reactions that may increase DOC photoreactivity in nature (Gao & Zepp, 1998; Voelker et al., 1997). A wide range of ionic and particulate transition metals (e.g., titanium oxide) have similar effects (Mariquit et al., 2008). Thus, DOC photoreactivity is not only affected by incoming solar radiation but also by chemical factors that influence the photoproduction of reactive oxygen species and other radicals.

Furthermore, water pH is a strong photoreactivity regulator (Figure 2). As pH decreases, organic molecules are increasingly protonated and molecular structures shrink to a compact form with strengthened molecular bonds, which increases absorption of UV light and boosts photoreactivity (Gennings et al., 2001). Moreover, interactions between iron, DOC, and UV light are favored by acidity, because iron is a better photocatalyst at low pH (Gu et al., 2017; Porcal et al., 2014). Interestingly, there are also reports of increasing photochemical processing of DOC at high pH and alkalinity (Reche et al., 1999), presumably caused by molecules expanding in deprotonated states leading to higher interception of light (Pace et al., 2012). Thus, DOC photoreactivity may have a U‐shaped relationship with pH, with the highest reactivities occurring at extreme pH values (Selvam et al., 2019). To conclude, any environmental property that increases the encounter rate of aquatic DOC with photons, protons, and reactive oxygen species positively affect photochemical DOC mineralization rates (Figure 2).

### Extrinsic controls on sorption and flocculation

Flocculation in the environment may be triggered by a specific compound, colloid, or particle surface, a so‐called coagulant or flocculant. Naturally occurring coagulants include metal cations, mineral particles, and positively charged polysaccharides or proteins. Long experience from treating drinking water (Matilainen et al., 2010) and wastewater (Teh et al., 2016) indicates that flocculants vary in their efficiency, compound specificity, and pH sensitivity. However, natural flocculation is generally favored by positively charged interaction interfaces occurring under acidic conditions. Hydrophobic DOC can also self‐flocculate and aggregate in response to decreasing pH (Colombo et al., 2015) or increasing salinity (Asmala et al., 2014). Owing to the combination of increased salinity and decreased water velocity, estuaries that receive DOC‐rich rivers are sites of abundant flocculation and precipitation that prevent 20%–40% of the DOC load from reaching coastal ecosystems (Lisitzin, 1994).

On mineral surfaces, the potential for DOC sorption is strongly linked to particle size. Indeed, clay has orders of magnitude more surface area per volume available for DOC interactions compared to larger particles (Mayer, 1994) and is also likely to stay suspended in the water column, increasing encounter probability. DOC sorption potential with surfaces is additionally affected by mineralogical properties, whereby iron oxide coatings form particularly stable bonds with DOC (Kleber et al., 2007; Saidy et al., 2013). In Swedish freshwater, DOC is susceptible to sorption, but there are usually insufficient surfaces to fulfill this capacity (Groeneveld et al., 2020).

Flocculation interacts strongly with photoreactivity since partial photooxidation of DOC is a major source of anionic organic acids that may in turn flocculate rapidly in reactions with positively charged surfaces (von Wachenfeldt et al., 2008). Moreover, flocculation rates are strongly positively correlated with biomineralization (von Wachenfeldt et al., 2009), presumably caused by bacterial release of sticky extracellular polysaccharides (Bhaskar & Bhosle, 2005; Decho, 1990) acting as strong flocculants (Shammi et al., 2017). Thus, considering these interactions, complete understanding of bulk DOC turnover in nature cannot be achieved without simultaneously considering bioreactions, photoreactions, and flocculation reactions and their intrinsic versus extrinsic controls.

## THE WAY FORWARD—INTRINSIC AND EXTRINSIC CONTROLS IN SYNCHRONY

**FIGURE 3:**
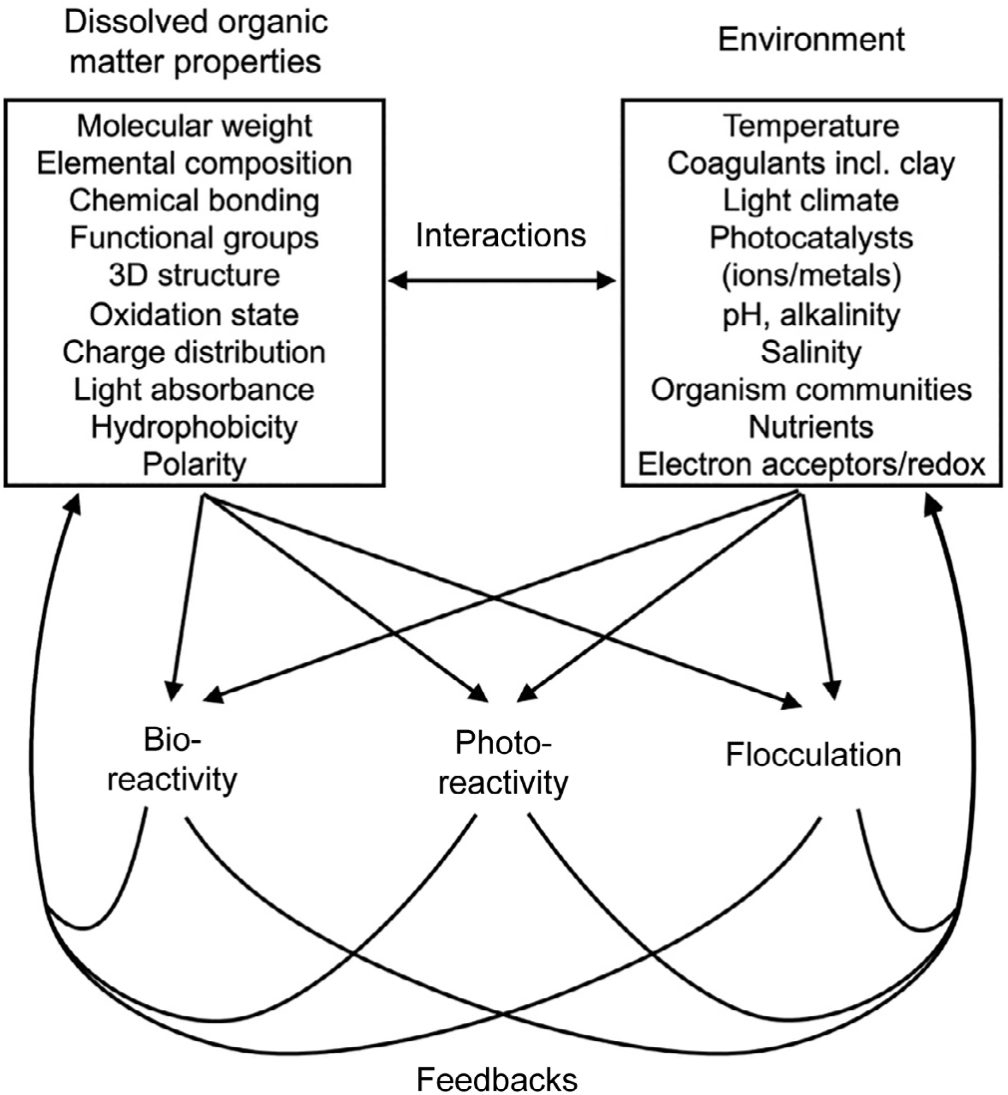
Conceptual regulation scheme, where arrows show how intrinsic (dissolved organic matter properties) and extrinsic (environment) factors together control different types of dissolved organic carbon reactivity, how they interact with each other and are affected by potential reaction feedbacks.

Our synthesis thus far illustrates that DOC reactivity is complex and regulated by different combinations of intrinsic and extrinsic factors that interact directly and via reaction feedback loops (Figure 3). Research on aquatic systems has only recently begun to approach both intrinsic and extrinsic dimensions of multiple organic carbon reaction types (Anderson et al., 2019), with examples mainly coming from the marine ecosystem modeling literature (Ge et al., 2020; Yakushev et al., 2017). However, given the rapid developments in molecular analytical methods (McCallister et al., 2018), quantifying reactive functional characteristics (Figure 1) should become increasingly feasible. This in turn will facilitate modeling the reaction rate of each functional group in specific environments (Figure 2).

To our knowledge, only one modeling study has attempted to predict DOC turnover in the aquatic continuum with explicit consideration of bioreactivity, photoreactivity, and flocculation while addressing some inherent organic matter properties by modeling aromatic and nonaromatic DOC separately (Anderson et al., 2019). Interestingly, in this study of large UK rivers, aromaticity had no effect on overall DOC turnover but caused a shift from biodegradation to photodegradation. Once DOC is discharged to the sea, photochemical reactions have the theoretical potential to remove all terrestrially derived organic molecules in coastal shelf seas (Aarnos et al., 2018), but the relative importance of different DOC turnover processes in the open sea remains to be modeled. Further development, application, and validation of this type of model in different existing and simulated future aquatic contexts is urgently needed and would be highly fruitful.

The most common framework to model DOC turnover under the joint influence of intrinsic and extrinsic controls is to use water residence time as the basis for predictions (e.g., Catalan et al. 2016). Water residence time is neither a physical property of the environment nor a property of DOC but integrates multiple intrinsic and extrinsic factors. For example, intrinsic DOC reactivity in freshwater landscapes generally decreases with increasing water residence time, as organic matter loses aromaticity (Weyhenmeyer et al., 2012) and becomes less susceptible to light and flocculation. Biological decay rates also decrease with residence time (Catalan et al., 2016), presumably because inherent bioreactive DOC is preferentially consumed. However, extrinsic factors may change systematically with increasing water residence times, in ways that can either boost or dampen reactivity (Creed et al., 2015; Selvam et al., 2019; Soares & Berggren, 2019). Thus, we propose a conceptual framework that recognizes that both intrinsic and extrinsic dimensions of DOC reactivity show patterns with water residence time.

Mechanistic models could supplement current efforts to understand the turnover of DOC in aquatic environments and predict future changes. In aquatic ecology, trait‐based mechanistic approaches are a rapidly developing field (Kiorboe et al., 2018). Here, the focus shifts from specific molecules in favor of their intrinsic traits, that is, which functional groups interact with extrinsic factors, and their impact on the DOC pool. Fundamental physical models of extrinsic drivers of reactivity may be easy to identify (e.g., Arrhenius equation), yet accounting for interactions with intrinsic DOC reactivity is challenged by chemical complexity. Approaching this complexity through molecular traits would considerably simplify it, for example, by studying simpler mixtures under controlled laboratory conditions. Thereafter, complexity could be slowly reintroduced as mechanistic understanding grows.

**FIGURE 4:**
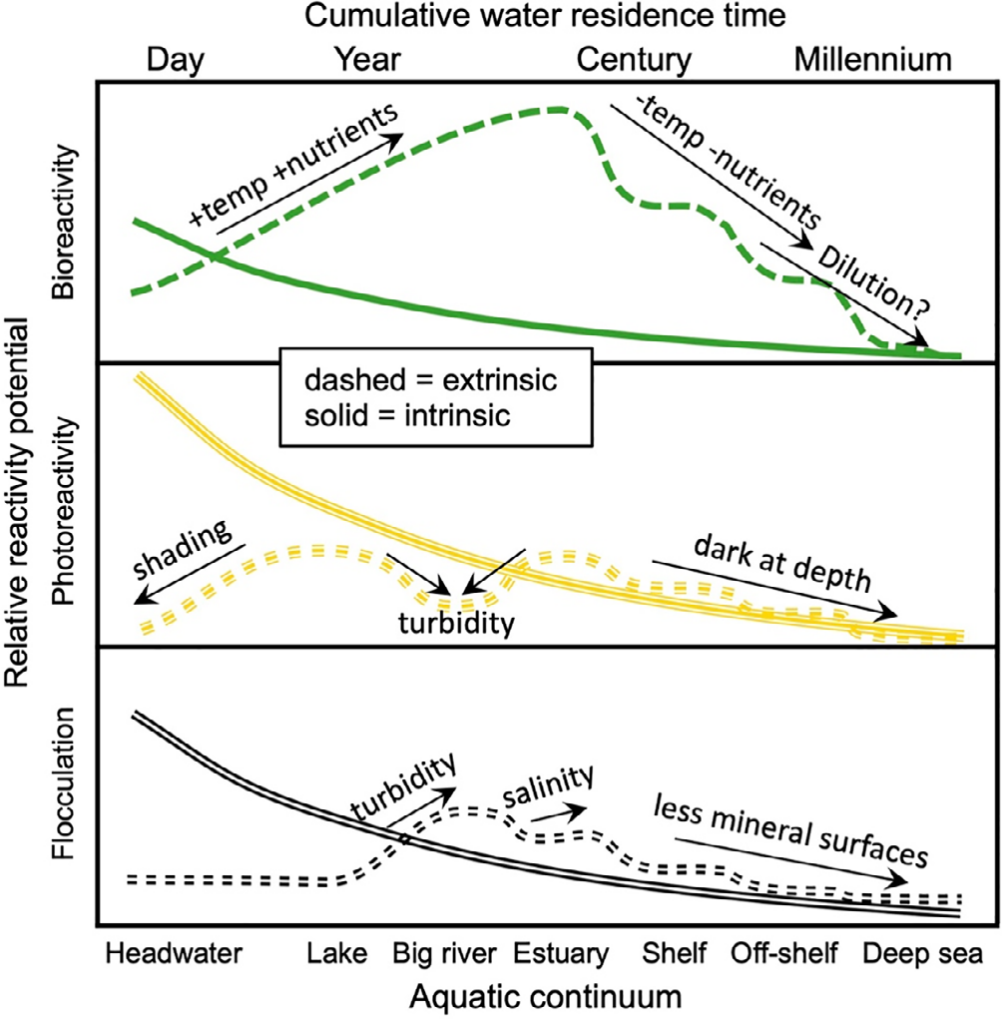
Hypothetical distribution of intrinsic (solid lines) and extrinsic (dashed lines) reactivity potential along the aquatic continuum and with increasing cumulative water residence times. Note that the shapes of the curves are highly generalized and that the true patterns are most likely different across localities and regions (especially in headwaters). Note that intrinsic reactivity is hypothesized to decrease smoothly with water residence time, whereas extrinsic reactivity may make discrete changes across ecosystem borders. Moreover, the intrinsic bioreactivity potential has intentionally been drawn at a relatively low level due to enzyme affinity limitations, but this is compensated by high extrinsic potential for biodegradation in the aquatic network.

Based on our framework, a suite of hypotheses has been formulated (Figure 4). Intrinsic organic matter properties are strongly modified by hydrological connectivity to specific landscape components in fast‐turnover headwaters (Coble et al., 2019; Tiwari et al., 2017), but with increasing downstream water residence time, chemical properties tend to converge (Creed et al., 2015; Massicotte et al., 2017) and reactive functional groups are lost (Weyhenmeyer et al., 2012). Thus, all intrinsic reactivity potentials from terrestrially derived DOC are to decrease during transit in the aquatic network (Figure 4). Conversely, the relative importance of extrinsic drivers of reactivity (Figure 2), such as light exposure, temperature, and nutrient supply, may increase from small, shaded headwaters to lakes and larger rivers (Soares & Berggren, 2019). Moreover, salinity will further boost flocculation once the estuaries are reached. However, extrinsic reactivity potentials should decrease again with the transition to marine systems because mineral particles and nutrients become scarce, temperature drops, and dilution potentially reduces substrate availability for bioreactions (Figure 4). It is therefore hypothesized that the highest DOC turnover rates will be expressed in systems with intermediate water residence times, where extrinsic potentials are relatively high and DOC still has partly intact inherent reactivity.

Current anthropogenic changes are systematically impacting DOC characteristics in freshwaters (Xenopoulos et al., 2021). For example, the ongoing widespread trends of increased terrestrially derived aromatics may (Berggren & Al‐Kharusi, 2020) or may not (Lapierre et al., 2013) decrease inherent bioreactivity in different regions, but photoreactivity (Anderson et al., 2019; Lapierre et al., 2013) and flocculation potential (Anderson et al., 2019) are expected to increase. From an extrinsic perspective, however, climate warming may strongly enhance biological DOC turnover owing to the higher Q_10_ of biological degradation. Thus, the contributions of different processes to the bulk DOC turnover in the future will depend on multiple global changes that affect both the inherent DOC properties and the environment where this DOC is processed.

## CONCLUSIONS AND OUTLOOKS

Now more than ever, an ability to accurately model the turnover and fate of DOC is critical to developing sound management strategies capable of addressing pressing environmental challenges. Simultaneous consideration of intrinsic and extrinsic controls on DOC reactivity opens up new avenues for research into DOC turnover in response to such changes and into effects on ecosystem services. Moreover, the distribution of intrinsic and extrinsic reactivity potentials in the aquatic continuum can provide new understanding of the relative importance of biological, photochemical, and flocculation reactions during transit from land to sea. We anticipate that models that simultaneously address different types of DOC reactions while considering both intrinsic and extrinsic controls of reactivity will be needed to take the research field to the next level.

## CONFLICT OF INTEREST

The authors declare no conflict of interest.
